# Contextualizing the standard maternal continuum of care in Pakistan: an application of revised recommendation of the World Health Organization

**DOI:** 10.3389/fpubh.2023.1261790

**Published:** 2024-01-11

**Authors:** Margubur Rahaman, Avijit Roy, Pradip Chouhan, Najma Iqbal Malik, Shamshad Bashir, Farooq Ahmed, Kun Tang

**Affiliations:** ^1^Department of Migration and Urban Studies, International Institute for Population Sciences, Mumbai, India; ^2^Department of Geography, Malda College, Malda, India; ^3^Department of Geography, University of Gour Banga, Malda, India; ^4^Department of Psychology, University of Sargodha, Sargodha, Pakistan; ^5^Department of Psychology, Lahore Garrison University, Lahore, Pakistan; ^6^Department of Anthropology, The Islamia University of Bahawalpur, Bahawalpur, Pakistan; ^7^Vanke School of Public Health, Tsinghua University, Beijing, China

**Keywords:** standard maternal continuum of care, predictors, fixed and random effect models, Fairlie decomposition analysis, Pakistan

## Abstract

**Objective:**

This study utilizes recent nationally representative data to contextualize the standard maternal continuum of care (SMCoC) in Pakistan. The revised SMCoC framework encompasses at least eight antenatal care visits, skilled birth attendants during delivery, and postnatal care within 48 h of childbirth.

**Methods:**

The study used a sample of 3,887 ever-married women aged 15–49 from the latest Pakistan Demographic and Health Survey (PDHS) conducted in 2017–18. Several statistical methods were employed: descriptive statistics, bivariate, multilevel logistic regression models, and Fairlie decomposition analysis.

**Results:**

Only 12% of women had accessed full SMCoC services in Pakistan. Education and the wealth quintile emerged as pivotal factors influencing the utilization of SMCoC. The likelihood of full SMCC utilization was more likely among higher educated women (OR: 3.37; 95% CI: 2.16–5.25) and those belonging to the wealthiest household wealth quintile (OR: 4.95; 95% CI: 2.33–5.51). Media exposure, autonomy, healthcare accessibility, residence, and region were also identified as significant predictors of SMCoC utilization among women.

**Conclusion:**

In conclusion, while most women did not utilize full SMCoC services in Pakistan, the pattern is substantially varied by background characteristics. Education, wealth quintile, mass media exposure, and autonomy were significant factors, along with geographical aspects such as healthcare accessibility and region. The study underscores the need for a multifaceted approach to ensure equitable access to full SMCoC services for women in Pakistan, addressing individual, socioeconomic, and geographical factors.

## 1 Introduction

In lower and middle-income countries (LMICs), the high maternal and neonatal mortality rates pose a significant public health concern (1). Notably, 94% of maternal deaths worldwide occur in LMICs (2). Within this group, Pakistan stands out with alarming maternal mortality (140 per 100,000 births) and neonatal mortality (42 deaths per 1,000 live births) rates (3). Consequently, in 2016, the World Health Organization (WHO) introduced a revised guideline known as the “Standard Maternal Continuum of Care (SMCoC)” aimed at reducing maternal and neonatal health vulnerabilities globally, with a particular emphasis on LMICs, including Pakistan (4). The updated SMCC guidelines encompass a comprehensive package, including a minimum of eight or more antenatal visits, skilled attendance during childbirth, and postnatal care provided by skilled healthcare professionals within 48 h of delivery (4). The revised SMCoC guideline strongly emphasizes antenatal visits, operating on the principle that increased antenatal visits lead to more systematic health screening during the antenatal period. The body of scientific evidence unequivocally supports the notion that increasing the frequency of antenatal visits holds the key to reducing maternal and child health vulnerability (5). Beyond its direct benefits, more antenatal visits also contribute to enhanced acceptance of skilled birth attendant deliveries and timely postnatal care, further fortifying the defenses against health vulnerabilities (6). Therefore, the revised SMCoC recommendation is more helpful in identifying and addressing pregnancy complications, assessing potential delivery risks, and preparing for post-delivery care (5). Despite several programs initiated by the Pakistani government in collaboration with international organizations to enhance maternal healthcare utilization, the utilization level still needs to improve in Pakistan (7). According to the latest Pakistan Demographic and Health Survey (PDHS) report for 2017–2018, only 51% of women received at least four antenatal care visits, 69% had skilled attendants during childbirth, and 62% received postnatal care within the first 48 h after delivery (3). Based on the previous SMCoC guidelines, the level of SMCoC utilization in Pakistan was 27% during 2012–13 (8). However, the current status of SMCoC utilization remains unexplored using the latest PDHS data. Therefore, the present study is relevant to understanding the current SMCoC utilization scenario in Pakistan.

Numerous studies have investigated the multifaceted landscape of SMCC utilization in Pakistan and elsewhere (8–11). The findings consistently underscore the critical role of socioeconomic poverty, lack of awareness, deficient healthcare infrastructure, and geographical remoteness as major determinants influencing the utilization of SMCoC services. However, it is essential to acknowledge that these studies relied on outdated data and measures, which have become less relevant over time. In light of the evolving understanding of maternal healthcare, this present study adopts the revised recommendations of SMCoC to construct its outcome variable (4), assessing the utilization of full SMCoC in Pakistan. The primary aim of this study is to provide a contemporary and comprehensive examination of the levels, patterns, and predictors of full SMCoC utilization in Pakistan. The study further explores urban–rural inequality in full SMCoC utilization in Pakistan. This study informs researchers and policymakers about maternal healthcare usage and its influencing factors in Pakistan. It will help researchers, healthcare authorities, and policymakers expand maternal health services coverage, addressing social and economic aspects and ultimately improving the country’s maternal and child health outcomes.

## 2 Materials and methods

The present study is based on secondary data from the latest Pakistan Demographic and Health Survey (PDHS) conducted during 2017–2018 (3). The survey gathered a wide range of demographic, socioeconomic, and health status from reproductive-aged women (15–49 years). The 2017–18 PDHS used a two-stage sample design, stratified into 16 groups by separating each of the eight regions into urban and rural areas. Implicit stratification and proportional allocation were applied by sorting the sampling frame and using probability-proportional-to-size selection. In the first stage, 580 clusters (enumeration blocks) were chosen with probabilities linked to household count. The second stage involved systematically selecting 28 households per cluster, totaling around 16,240 households, and successfully interviewing 12,364 women aged 15–49 years (3). Household selection occurred centrally at the National Institute of Population Studies (NIPS) data processing office, with no replacements or changes to pre-selected households to prevent bias. Weighting factors were calculated and applied to ensure national representativeness for Pakistan, including FATA and ICT Islamabad. The current study included a total weighted sample of 3,887 women aged 15–49 years to investigate the context, trends, and determinants of SMCoC utilization in Pakistan. The sample is limited to women with the most recent live births within the 2 years preceding the survey. The present study restricted the sample to women with the most recent live births within the 2 years preceding the survey to match the postnatal care information in the PDHS (3). The study excludes samples from Pakistan Azad Kashmir and Gilgit Baltistan to adhere to PDHS guidelines and provide nationally representative insights into SMCoC utilization.

### 2.1 Outcome variable

The key outcome variable of the study was the full utilization of the standard maternal continuum of care (SMCoC). Following the latest WHO recommendation, the SMCoC includes the utilization of a minimum of eight antenatal care (ANC) appointments, skilled birth attendant (SBA) delivery, and postnatal care (PNC) within 48 h after delivery (4). The utilization of these three healthcare services was coded as 1, indicating “full SMCoC utilization,” and otherwise coded as 0, indicating “non-utilization or partial utilization of SMCoC.”

### 2.2 Explanatory variables

The present study included a range of explanatory variables based on a systematic literature review (8, 12). The selected explanatory variables were the respondent’s age, education level, last birth planning status, household wealth quintile, health insurance, healthcare autonomy, frequency of mass media exposure, healthcare accessibility, place of residence, and region. The details of the explanatory variables are included in Supplementary Table S1.

### 2.3 Statistical analysis

A range of analyses are applied to accomplish study objectives. Descriptive statistics were employed to present the background of the sample, including a weighted frequency distribution with a 95% confidence interval (CI). Bivariate analysis was used to illustrate the weighted distribution of full SMCoC utilization alongside background characteristics. The Pearson chi-square (χ^2^) test was also conducted to assess the independence of two variables. Lastly, a multilevel logistic regression analysis was applied to identify potential factors influencing full SMCoC utilization in Pakistan and to analyze the variation of predictors across different sampling units (districts, clusters, and households). A four-level random intercept logistic regression model was utilized in this investigation. The details of the multilevel model are included in the Supplementary File S1. The PDHS dataset has a hierarchical structure (districts, clusters, households, and individuals), making it suitable for a hierarchical regression model (multilevel random intercept logistic regression model). Before conducting the multilevel analysis, the study assessed potential multicollinearity among the explanatory variables using the variance inflation factor (VIF) and found no indications of multicollinearity issues. Further, the present study performed the Fairlie decomposition model to contextualize the urban–rural gap in the utilization of full SMCoC. The Blinder-Oaxaca decomposition method has been extensively used in economics and health to identify and measure differences between two groups (13–15). The details of the Fairlie decomposition model are included in Supplementary File S2. All statistical analyses were performed using Stata version 17.0 (StataCorp LP, College Station, TX, United States).

## 3 Results

### 3.1 Background characteristics of the study population

Out of the total weighted study sample of 3,887 women aged 15–49 years, more than half of the respondents belonged to the age groups 20–24 (25.9%; 95% CI: 24.5–27.3) and 25–29 (31.7%; 95% CI: 30.2–33.2). About 86% of women reported that their last birth was planned (86.5%; 95% CI: 85.4–87.6). Almost half (47.3%; 95% CI: 45.7–48.9) of women had no formal education (Table 1). More than 50% of women had no healthcare autonomy (56.6%; 95% CI: 55.1–58.2) and had moderate mass media exposure (55.8%; 95% CI: 54.3–57.4). Only one-fifth of the respondents belonged to the richest wealth quintile (19.3%; 95% CI: 18.1–20.6). Similarly, the health insurance coverage was meager (1.2%; 95% CI: 1.1–1.6). Most women resided in rural areas (67%; 95% CI: 65.5–68.4) and reported easily accessible healthcare facilities (54.3%; 95% CI: 52.7–55.9). Most participants were from the Punjab region (52.7%; 95% CI: 51.1–54.3), followed by Sindh (23.2%; 95% CI: 21.9–24.6).

**Table 1:** Background characteristics of the study population, Pakistan, Pakistan Demographic Health Survey, 2017–18.

Background characteristics	Weighted sample (*n*)	Weighted percentage	95% CI
Total weighted sample (N)	3,887		
**Age group**			
15–19	223	5.7	5.0–6.5
20–24	1,005	25.9	24.5–27.3
25–29	1,231	31.7	30.2–33.2
30–34	887	22.8	21.5–24.2
35–39	541	13.9	12.9–15
**Last birth planning status**			
Planned	3,363	86.5	85.4–87.6
Unplanned	524	13.5	12.4–14.6
**Educational attainments**			
No schooling	1,838	47.3	45.7–48.9
Primary	613	15.8	14.6–16.9
Secondary	882	22.7	21.4–24.0
Higher	554	14.3	13.2–15.4
**Frequency of mass media exposure**			
Low	1,475	38.0	36.4–39.5
Moderate	2,169	55.8	54.3–57.4
High	243	6.2	5.5–7.0
**Healthcare autonomy**			
No	2,201	56.6	55.1–58.2
Yes	1,686	43.4	41.8–44.9
**Household health quintile**			
Poorest	836	21.5	20.2–22.8
Poorer	742	19.1	17.9–20.3
Middle	834	21.5	20.2–22.8
Richer	723	18.6	17.4–19.9
Richest	752	19.3	18.1–20.6
**Health insurance beneficiary**			
No	3,841	98.8	98.4–99.1
Yes	46	1.2	1.1–1.6
**Healthcare accessibility**			
Difficult	1,776	45.7	44.1–47.3
Easily accessible	2,111	54.3	52.7–55.9
**Place of residence**			
Urban	1,285	33.0	31.6–34.5
Rural	2,602	67.0	65.5–68.4
**Region**			
Punjab	2,049	52.7	51.1–54.3
Sindh	903	23.2	21.9–24.6
Khyber Pakhtunkhwa	620	15.9	14.8–17.1
Baluchistan	197	5.1	4.4–5.8
ICT Islamabad	31	0.8	0.6–1.1
Federally Administered Tribal Areas	87	2.2	1.8–2.8

CI, Confidence Interval.

### 3.2 Levels of SMCC utilization with background characteristics

Only 12% of the respondents utilized the full SMCoC in Pakistan during the study period (Table 2). However, the level of full SMCoC utilization was comparatively low among women aged 15–19 (70%; 95% CI:4.8–11.9) or who had no formal education (3.3%; 95% CI: 2.6–4.2) or belonged to the poorest wealth quintile (2.1%; 95% CI: 1.3–3.4). Similarly, the level of full SMCoC utilization was lower among the women with low mass media exposure (3.4%; 95% CI: 2.6–4.4) than their counterparts. The utilization of full SMCoC was considerably higher among women living in urban (21.7%; 95% CI: 19.5–24.1) than rural (7.5%; 95% CI: 6.6–8.6) counterparts. Among the geographical regions, full SMCC utilization was lowest in Baluchistan (1.6%; 95% CI: 1.3–4.7).

**Table 2:** Prevalence of Standard Maternal Continuum of Care (SMCoC) utilization by selected background characteristics of women aged 15–49 years, Pakistan, Pakistan Demographic Health Survey, 2017–18.

Background characteristics	Prevalence	95% CI	Chi2 *p*-value
Total	12.2	11.2–13.3	
**Age group**			
15–19	7.6	4.8–11.9	0.001
20–24	10.8	9.0–12.9	
25–29	14.0	12.2–16.1	
30–34	15.1	12.9–17.7	
35–39	7.8	5.8–10.4	
**Last birth planning status**			
Planned	12.0	10.9–13.1	
Mistimed	13.6	11.7–16.8	0.001
**Educational attainments**			
No schooling	3.3	2.6–4.2	
Primary	10.2	8.1–12.9	0.001
Secondary	19.3	16.8–22	
Higher	32.6	28.9–36.7	
**Frequency of mass media exposure**			
Low	3.4	2.6–4.4	
Moderate	16.5	15.0–18.2	0.001
High	27.4	22.1–33.4	
**Healthcare autonomy**			
No	9.0	7.8–10.2	0.001
Yes	16.4	14.8–18.3	
**Household health quintile**			
Poorest	2.1	1.3–3.4	
Poorer	2.7	1.7–4.1	
Middle	8.1	6.4–10.2	0.001
Richer	20.8	18.0–23.9	
Richest	29.1	26–32.5	
**Health insurance beneficiary**			
No	12.2	11.2–13.2	0.001
Yes	15.8	7.8–29.5	
**Healthcare accessibility**			
Difficult	8.2	7.0–9.5	
Easily accessible	15.6	14.1–17.2	0.001
**Place of residence**			
Urban	21.7	19.5–24.1	0.001
Rural	7.5	6.6–8.6	
**Region**			
Punjab	15.0	13.6–16.7	
Sindh	13.9	11.8–16.3	0.001
Khyber Pakhtunkhwa	4.2	2.9–6.1	
Baluchistan	1.6	1.3–4.7	
ICT Islamabad	34.9	20.2–53.1	
Federally Administered Tribal Areas	0.8	0.1–7.9	

CI, Confidence Interval.

### 3.3 Results from multilevel logistic regression

The results of the multilevel regression analysis are presented in Table 3, which highlights the influence of fixed-effect and random-effect factors. The intra-class correlation coefficient (ICC) demonstrated that household differences account for 43.4% of the overall variability in full SMCoC utilization, followed by clusters (19.3%) and districts (6%). The log-likelihood ratio test (LR) vs. logistic regression has a *p*-value of 0.045 in the random effect section. The result displayed a considerable difference across the household, cluster, and district levels for selected indicators of SMCoC utilization in Pakistan (Table 3).

**Table 3:** Adjusted odds of Standard Maternal Continuum of Care (SMCoC) utilization among women 15–49 years, Pakistan, Pakistan Demographic and Health Survey, 2017–18.

Background characteristics	Adjusted OR	*p*-value	95% CI
**Age group**			
15–19			
20–24	0.81	0.480	0.45–1.45
25–29	0.85	0.568	0.48–1.50
30–34	0.82	0.517	0.46–1.49
**35–39**	**0.67**	**0.036**	**0.55–0.89**
**Last birth planning status**			
Planned			
Mistimed	**0.89**	**0.050**	**0.85–0.93**
**Educational attainments**			
No schooling			
Primary	1.33	0.215	0.85–2.09
Secondary	**1.98**	**0.001**	**1.33–2.95**
Higher	**3.37**	**0.001**	**2.16–5.25**
**Frequency of mass media exposure**			
Low			
Moderate	**2.02**	**0.001**	**1.37–2.98**
High	**2.06**	**0.006**	**1.23–3.45**
**Healthcare autonomy**			
No			
Yes	**1.29**	**0.050**	**1.19–1.37**
**Household health quintile**			
Poorest			
Poorer	1.17	0.673	0.57–2.40
Middle	1.85	0.082	0.93–3.68
Richer	**3.14**	**0.002**	**1.54–6.38**
Richest	**4.95**	**0.001**	**2.33–5.51**
**Health insurance beneficiary**			
No			
Yes	2.40	0.062	0.96–6.02
**Healthcare accessibility**			
Difficult			
Easily accessible	0.90	0.474	0.68–1.19
**Place of residence**			
Urban			
Rural	**0.90**	**0.005**	**0.87–0.97**
**Region**			
Punjab			
Sindh	1.42	0.115	0.92–2.20
Khyber Pakhtunkhwa	**0.44**	**0.002**	**0.26–0.74**
Baluchistan	**0.29**	**0.001**	**0.14–0.60**
ICT Islamabad	**2.36**	**0.040**	**1.95–2.89**
Federally Administered Tribal Areas	**0.26**	**0.020**	**0.08–0.80**
**Constant**	**0.02**	**0.001**	**0.01–0.05**
**Random intercept parameters**			
Var (Districts)	0.40		0.23–0.77
Var (Cluster)	0.72		0.47–1.10
Var (Households)	1.40		0.76–2.58
**Wald chi2(23) = 143.41, chi2(3) = 31.32, Prob > chi2 = 0.045**			
ICC (Districts)	0.06		0.04–0.12
ICC (Clusters)	0.19		0.15–0.24
ICC (Households)	0.43		0.33–0.54

CI, Confidence Interval; OR, Odds Ratio. Bold values shows they are important values.

The likelihood of full SMCoC utilization was 33% (OR: 0.67; 95% CI: 0.55–0.89) less likely among women aged 35+ years (Table 3). The level of education and household wealth quintile was positively associated with full SMCoC utilization. In particular, the odds of full SMCoC utilization were 3.8 times higher among higher-educated women (OR: 3.37; 95% CI: 2.16–5.25) than the reference group, i.e., women with no formal education. Similarly, the likelihood of SMCoC utilization was almost five times higher among the richest wealth quintile group (OR: 4.95; 95% CI: 2.33–5.51) than the poorest counterparts. Women’s mass media exposure and healthcare autonomy were also found to be significant predictors of full SMCoC utilization. The women with high mass media exposure were 2.26 (OR: 1.26; 95% CI: 1.23–3.45) times more likely to utilize the full SMCoC service than the reference category, i.e., the low mass media exposure group. Similarly, women with healthcare autonomy were 29% (OR: 1.29; 95% CI: 1.19–1.37) more likely to utilize the fullSMCoC service than their counterparts. The likelihood of full SMCoC utilization was 10% (OR: 0.90; 95% CI: 0.87–0.97) less likely in rural areas than urban counterparts. Regarding geographical region, the likelihood of full SMCC utilization was found to be significantly lower in the Baluchistan region (OR: 0.29; 95% CI: 0.14–0.60) and the Federally Administered Tribal Areas (FATA) (OR: 0.26; 95% CI:0.08–0.80) than in the reference category, i.e., in the Punjab region (Table 3). Adopting the older version of the WHO recommendations for full SMCoC, the patterns of adjusted odds of full SMCoCutilization were also almost the same (Supplementary Table S3).

### 3.4 Results from Fairlie decomposition analysis

Table 4 denotes the decomposition results to explain the gap in the utilization of full SMCoC between urban and rural women. The proportion of full SMCoC utilization among urban women was 0.29, considerably higher than among rural women (0.11). The results also revealed that the factors included in the study explained about 83% of the gap in the prevalence of full SMCoC between urban and rural women.

**Table 4:** Decomposition result of the urban–rural difference in utilization in SMCoC in Pakistan, Pakistan Demographic Health Survey, 2017–18.

	Utilization of SMCoC
Proportion urban	0.29
Proportion rural	0.11
Difference in proportion (urban–rural)	0.18
Total proportional explained	0.15
Percentage explained	83.3

Table 5 presents the effect and contribution of each predictor variable in the urban–rural difference in the utilization of full SMCoC. Results indicate that the household wealth quintile was the key predictor, explaining about 60% of the difference in utilization of full SMCoC between urban and rural, followed by women’s education (24%) and distance to healthcare access (7%). Similarly, mass media exposure and women’s healthcare autonomy significantly contributed to widening the gap in the utilization of full SMCoC.

**Table 5:** Effect and contribution of each predictor variable in the urban–rural difference in utilization of SMCoC in Pakistan, Pakistan Demographic Health Survey, 2017–18.

	Coefficient	% contribution
Woman’s age	0.001	0.5
Last birth planning status	−0.001	−0.4
Women’s education level	0.036***	24.2
Mass media exposure	0.005***	3.5
Healthcare autonomy	0.004***	3
Household wealth quintile	0.089***	59.8
Health insurance coverage	0.002	1.2
Healthcare accessibility	0.011**	7.1
Geographical region	0.002*	1.1

Level of significance: *p < 0.05, **p < 0.01, ***p < 0.001.

**Figure 1:**
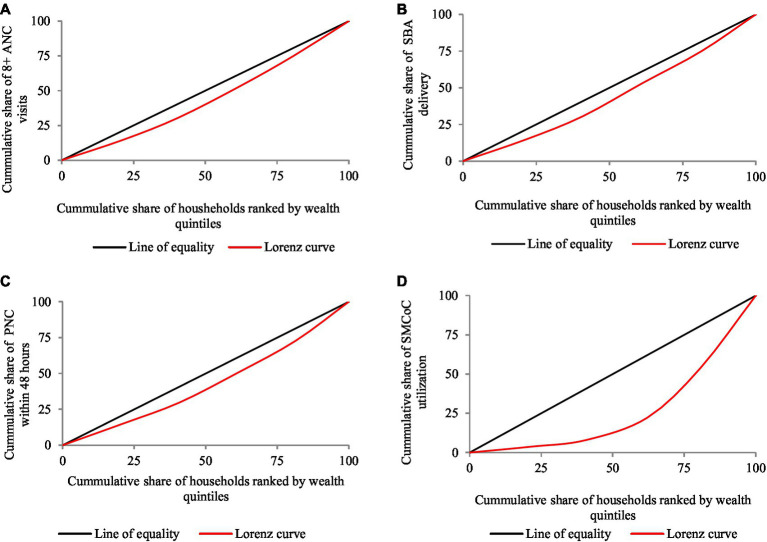
Concentration curve showing the wealth-based inequality in utilization of **(A)** 8+ ANC, **(B)** SBA delivery, **(C)** PNC and **(D)** SMCoC among reproductive aged women in Pakistan, Pakistan Demographic Health Survey, 2017–18.

We further estimate wealth-based inequalities in the utilization of SMCoC and vice versa (Figure 1). Figures 1A–C reveal that standard ANC, SBA, and PNC visits are concentrated among women of reproductive age from poor socioeconomic strata. Likewise, Figure 1D displays that utilization of SMCoC is concentrated among women of reproductive age among poor socioeconomic strata.

## 4 Discussion

The underutilization of the SMCoC and its association with maternal and child mortality remain significant public health concerns worldwide, particularly in Low- and Middle-Income Countries (LMICs) (2). The WHO has revised SMCoC guidelines, emphasizing the importance of a minimum of 8 antenatal care (ANC) visits, replacing the previous recommendation of 4 or more ANC visits. The revised SMCoC guidelines also include the utilization of skilled birth attendants during delivery and postnatal care (PNC) within 48 h of the delivery as essential components. SMCoC, endorsed by the WHO, is a crucial strategy for promoting the health of pregnant women (4). Prior studies have shown that the number of antenatal care visits is vital for subsequent healthcare utilization. By adhering to SMCoC, LMICs have the potential to reduce the burden of maternal and child health vulnerabilities (5). Therefore, this study has adopted the revised WHO SMCoC guidelines to assess maternal healthcare utilization in Pakistan. The study findings indicate that most women (88%) failed to utilize full SMCoC in Pakistan. The timing of the announcement of the revised WHO recommendation, specifically in 2016–17, coincided with the survey period of the latest PDHS in the same timeframe (3). Consequently, the findings in the present study revealed a notably low level of full SMCoC. While the WHO advocated for the revision of guidelines, it is essential to recognize that the implementation of the revised program and its anticipated outcomes require a significant amount of time. In this perspective, the present study can be considered a foundational investigation, serving as a baseline for subsequent research. It paves the way for assessing how the levels and predictors of full SMCoC utilization, guided by the WHO from 2016, evolve over time. Further, a longitudinal perspective using an upcoming series of PDHS data is crucial for gaining insights into the dynamics and progress in maternal healthcare practices in Pakistan. However, it is worth noting and acknowledging the commendable progress that Pakistan achieved regarding SMCoC utilization between 2007 and 2017 (Supplementary Table S2) when applying the outdated version of the SMCoC (details of the outdated recommendation available in Supplementary File S3). Similarly, although socioeconomic and spatial variation exists in SMCoC utilization in Pakistan, the socioeconomic and spatial disparity substantially decreased during 2014–2017 (8).

Notwithstanding the commendable progress made in increasing the coverage of full SMCoC over the last decade, the level of full SMCoC was observed as 12% according to the revised WHO guidelines (2016) (Table 2) and 40% according to the outdated guidelines (2001) (Supplementary Table S2). The impact of low SMCoC utilization is evident in the observed high maternal and child mortality rates in Pakistan compared to its neighboring country, India (8, 9). Of particular concern is the Neonatal Mortality Rate (NNM), which stood at 42 deaths per 1,000 live births in 2017, reflecting a decrease of 12 points from 2007 when the rate was 54 deaths per 1,000 live births (3). The Maternal Mortality Rate (MMR) in Pakistan in 2015 was reported as 178 deaths per 100,000 live births, marking a significant reduction of 75 points from 2007 when the rate was 253 deaths per 100,000 live births (3, 4). However, by 2019, Pakistan, classified as one of the low-income countries, reported a high MMR of 186 deaths per 100,000 live births, indicating a 32% increase from 2017 (140 deaths per 100,000 live births). Despite the WHO’s recommendation of the revised version of SMCoC in 2016, the Pakistani government continues to follow the old recommendation of SMCC (4). The revised version of SMCoC (2019) recommends at least eight antenatal care (ANC) visits instead of the previous recommendation of at least four ANC visits, aiming to enhance maternal health screening and care and reduce the risk of maternal and child mortality. Therefore, the current study underscores the urgent need to implement the WHO’s revised recommendation of SMCoC (2016) in Pakistan to address the increasing trend in MMR observed during 2017–19.

In line with prior research (8, 9, 11, 16), this study found women’s age, educational attainment, household wealth, exposure to mass media, and healthcare autonomy as significant factors in accessing SMCoC services in Pakistan. Consistent with earlier studies (8, 16), it is evident that women aged 35 and older were less likely to utilize the SMCoC services than their younger counterparts. The issue of underutilization among older women may be attributed to factors such as lower levels of education, limited health awareness, and a preference for traditional modes of maternal care. However, a more comprehensive study is needed to fully understand the nexus between women’s age and SMCoC utilization. In resonance with prior research (8, 16, 17), the present study emphasizes the positive association between women’s education and SMCoC utilization. Existing literature suggests that education equips women with enhanced health awareness, fostering a greater propensity to seek and receive appropriate healthcare, consequently contributing to improved overall well-being (17, 18). Moreover, educated women often engage in improved jobs and have greater autonomy, and together, these factors synergistically promote the utilization of SMCoC services (19–21). In essence, this study reinforces the integral role of education in shaping women’s healthcare decisions, demonstrating the cascading effects of education on health awareness, autonomy, and socioeconomic status—all of which intricately influence the utilization of vital SMCoC services. Like women’s education, women’s exposure to mass media and healthcare autonomy are also positively associated with SMCC utilization. These findings align with numerous previous studies (20, 22, 23), suggesting that mass media is crucial in enhancing knowledge about the benefits of maternal healthcare utilization, diverse sources of healthcare facilities, and ongoing government maternal health programs. Additionally, healthcare autonomy empowers women to access healthcare facilities on time and allows them to make choices based on accessibility.

In addition to socio-demographic factors, the household wealth quintile was a significant driver of SMCoC utilization in this study. Previous studies have also shown a positive association between economic poverty and the underutilization of maternal healthcare services (24, 25). Existing literature highlights that economic deprivation creates barriers to accessing private maternal healthcare services, often resulting in out-of-pocket expenditures related to health check-ups, transportation, and medication acquisition costs (25, 26). Notably, this study found that less than 2% of respondents had health insurance, and there was no significant association between health insurance and maternal healthcare utilization. This lack of significance may be attributed to Pakistan’s low insurance coverage. Nonetheless, the association between insurance and SMCoC utilization could be more significant, highlighting the need to address Pakistan’s inadequate health insurance coverage. The present study suggests increasing insurance coverage in Pakistan by introducing affordable schemes by private insurance companies or publicly subsidized insurance programs.

Similar to many previous studies (8, 16, 27, 28), the current study identified that place of residence and region are significantly associated with SMCoC utilization. Respondents who often faced difficulties reaching healthcare facilities, living in rural areas, or residing in Baluchistan are more likely to report underutilization of SMCoC. The research findings highlight the significance of women’s education and household wealth quintile as key contributors to urban–rural inequality in SMCoC. Therefore, placing a strong emphasis on advancing women’s education and enhancing household well-being in rural Pakistan can play a pivotal role in mitigating the underutilization of maternal healthcare services. Consequently, this study recommends developing strategies to address geographical inequalities (regional, district, cluster, and household level) in SMCoC utilization in Pakistan, with a particular focus on rural and underserved regions. Such measures can enhance maternal healthcare access, especially in areas grappling with facility inadequacy, ultimately advancing maternal well-being nationwide.

### 4.1 Strengths and limitations

To the best of our knowledge, this study represents a pioneering cross-sectional investigation, uniquely employing the revised guidelines for SMCoC recommended by the WHO. This revised approach and robust statistical analyses strengthen the findings by examining the predictors of SMCoC utilization. Additionally, applying multilevel regression provided the different sampling unit level variations in predictors of SMCoC in Pakistan. Finally, the study provides a comprehensive understanding of SMCoC utilization in the country using the latest PDHS data.

Nevertheless, while the study boasts several strengths, the study also acknowledges several limitations. Firstly, the data on maternal healthcare services utilization relied on respondents’ self-reporting, which may be susceptible to over-reporting and underreporting biases. Secondly, the cross-sectional nature of the survey data prevents us from establishing causality between the outcomes and explanatory variables. Thirdly, the study solely focuses on married women, which limits its ability to assess healthcare utilization among unmarried women. Nevertheless, it is important to note that the practice of childbirth without marriage in Pakistan is negligible. Lastly, this study is primarily quantitative, which limits our ability to explore the qualitative drivers of healthcare access, such as traditional practices, beliefs, cultural norms, and habits. Despite these limitations, this study contributes valuable insights into the factors influencing SMCoC utilization in Pakistan, shedding light on improvement in maternal healthcare services and providing a foundation for future research endeavors that may delve deeper into the qualitative aspects of healthcare access.

## 5 Conclusion

In summary, the study underscores the presence of significant variations in accessing full SMCoC services across the socioeconomic and demographic groups, as well as by place of residence and region in Pakistan. Although most women did not utilize full SMCoC services in Pakistan, it is evident that certain factors play a pivotal role in utilizing the services. Specifically, the study found that women’s age, level of education, exposure to mass media, and healthcare autonomy are key individual-level predictors of SMCoC utilization. This highlights the urgency of policy measures to empower women, thus addressing the underutilization of SMCoC services. The pronounced wealth-based inequality in full SMCoC utilization further emphasizes the need for targeted efforts through pro-poor health programs and policy interventions. Geographical factors, such as healthcare accessibility, place of residence, and region, also emerge as significant predictors of SMCoC utilization. Therefore, it is imperative to incorporate geographical considerations into strategies to achieve spatial equality in full SMCoC utilization throughout Pakistan. Additionally, our multilevel model revealed substantial variations at the district, cluster, and household levels in the predictors of SMCoC utilization. The finding underscores the importance of adopting a more spatially informed and population-centric approach to ensure comprehensive SMCoC coverage for all population segments. In conclusion, the study highlights the complexity of factors influencing SMCoC utilization and calls for a multifaceted approach that addresses individual, socioeconomic, and geographical determinants to ensure equitable access to these vital services for women across Pakistan.

## Data availability statement

Publicly available datasets were analyzed in this study. This data can be found at: https://dhsprogram.com/data/available-datasets.cfm.

## Author contributions

MR: Conceptualization, Formal analysis, Investigation, Methodology, Validation, Visualization, Writing – original draft. AR: Conceptualization, Data curation, Formal analysis, Investigation, Methodology, Writing – original draft. PC: Data curation, Validation, Visualization, Writing – original draft. NM: Software, Validation, Visualization, Writing – review & editing. SB: Investigation, Validation, Visualization, Writing – review & editing. FA: Funding acquisition, Supervision, Validation, Visualization, Writing – original draft, Writing – review & editing. KT: Funding acquisition, Validation, Visualization, Writing – review & editing.
