# Supplementary material for: Contextualizing the standard maternal continuum of care in Pakistan: an application of revised recommendation of the World Health Organization
Source: Front Public Health. 2024 Jan 11;11:1261790. doi: 10.3389/fpubh.2023.1261790 (PMC10809265; doi:10.3389/fpubh.2023.1261790)
Supplement: Supplementary file 1 [file Data_Sheet_2.docx]

**Supplementary File S1**

**Multilevel analysis**

The Four-level random intercept logistic model has been selected for the likelihood of the SMCoC utilization (*i*) in the HH *j*, cluster*k*, and district *l* being SMCoC utilization (Ƴ*_ijkl_*= 1).

$$logit\left( \pi_{ijlk} \right)=\beta_{o}+{BX}_{ijkl}+(f_{0k}+m_{0jk}+p_{0jkl}+s_{0ijkl})$$

This model calculates the log odds of $\pi_{ijlk}$ adjusted for vector$X_{ijkl}$ of predictor variables assessed at the individual level. The parameter $\beta_{o}$indicates the reference category of all variables with log odds of the SMCoC utilization. The random effect within the parentheses is measured as a residual differential for the district *l* (*f_0l_*), cluster*k* (*m_0kl_*), HH *j* (*p_0jkl_*), and individual *i* (*s_0ijlk_*) considered to be independent and normally distributed with mean 0 and variance $\sigma_{f0}^{2}$, $\sigma_{m0}^{2}$, $\sigma_{p0}^{2}$, and $\sigma_{s0}^{2}$, respectively. The variances quantify between districts, between PSU, and between household variations, respectively, in the log-odds of the SMCoC utilization. The results of multilevel logistic regression are presented in terms of adjusted odds ratios (AORs).
