# Supplementary material for: Contextualizing the standard maternal continuum of care in Pakistan: an application of revised recommendation of the World Health Organization
Source: Front Public Health. 2024 Jan 11;11:1261790. doi: 10.3389/fpubh.2023.1261790 (PMC10809265; doi:10.3389/fpubh.2023.1261790)
Supplement: Supplementary file 2 [file Data_Sheet_3.docx]

**Supplementary File S2**

**Fairlie decomposition analysis**

This method uses ordinary least squares to decompose the disparity in mean outcomes between two groups by utilizing their additive separability. However, this method is not applicable when the outcome variable is binary, which is the case for our model in this paper. To overcome this limitation, the present study utilized a binary-model-appropriate version of the Blinder-Oaxaca approach, developed by Fairlie [14], to decompose the urban-rural disparity in the utilization of SMCoC. In contrast to the Blinder-Oaxaca approach, the Fairlie technique decomposes the difference between the mean of projected probabilities rather than the mean of outcomes. The Fairlie decomposition method utilizes Logit regression estimates, and we used the Fairlie package, which supports non-linear decomposition for binary dependent variables. The decomposition for a non-linear equation 𝑦 = (𝑥𝛽) can be written as:

$${ȳ}^{o}-\bar{y}^{s}= \left[ \sum_{i=1}^{N^{o}} \frac{F\left( x_{i}^{o}\beta^{o} \right)}{N^{o}}-\sum_{i=1}^{N^{s}} \frac{F\left( x_{i}^{s}\beta^{o} \right)}{N^{s}} \right]+\left[ \sum_{i=1}^{N^{o}} \frac{F\left( x_{i}^{s}\beta^{o} \right)}{N^{s}}-\sum_{i=1}^{N^{s}} \frac{F\left( x_{i}^{s}\beta^{s} \right)}{N^{s}} \right]$$

Where N*^J^* is the sample size for interest group *j*. *y^j^*is the average probability of the binary outcome of the interest group *j* and *F* is the cumulative distribution function from the logistic distribution. Here, superscripts *O* and *S* stand for rural and urban.

Using coefficient estimates from a logit regression for a pooled sample, the independent contribution of x_1_ to the group gap can then be expressed as:

$$\frac{1}{N^{s}}\sum_{i=1}^{N^{s}} F\left( \hat{\alpha}+x_{1_{i}}^{O}\hat{\beta}_{1}^{*}+x_{2_{i}}^{O}\hat{\beta}_{2}^{*} \right)-F\left( \hat{\alpha}+x_{1_{i}}^{s}\hat{\beta}_{1}^{*}+x_{2_{i}}^{0}\hat{\beta}_{2}^{*} \right)$$

Similarly, the gap due to x_2_ can be expressed as:

$$\frac{1}{N^{s}}\sum_{i=1}^{N^{s}} F\left( \hat{\alpha}+x_{1_{i}}^{s}\hat{\beta}_{1}^{*}+x_{2_{i}}^{O}\hat{\beta}_{2}^{*} \right)-F\left( \hat{\alpha}+x_{1_{i}}^{s}\hat{\beta}_{1}^{*}+x_{2_{i}}^{s}\hat{\beta}_{2}^{*} \right)$$
