# Supplementary material for: Contextualizing the standard maternal continuum of care in Pakistan: an application of revised recommendation of the World Health Organization
Source: Front Public Health. 2024 Jan 11;11:1261790. doi: 10.3389/fpubh.2023.1261790 (PMC10809265; doi:10.3389/fpubh.2023.1261790)
Supplement: Supplementary file 3 [file Data_Sheet_4.docx]

**Supplementary File S3**

**Mandated services under the Standard Maternal Continuum of Care (SMCoC)** recommended by the World Health Organization (revised vs. non revised) [2]

| **Older version of SMCoC components** | **Latest version of SMCoC components** |
| --- | --- |
| At least 4 ANC (ANC 4+) + Skilled Birth Attendant delivery + Post natal care within 48 hours of delivery. | At least 8 ANC (ANC 8+) + Skilled Birth Attendant delivery + Post natal care within 48 hours of delivery. |
